# A hybrid fuzzy-AHP-TOPSIS model for evaluation of manufacturing relocation decisions

**DOI:** 10.1007/s12063-022-00284-6

**Published:** 2022-06-30

**Authors:** Movin Sequeira, Anders Adlemo, Per Hilletofth

**Affiliations:** 1 Department of Industrial Product Development Production and Design, Jönköping University, 551 11 Jönköping, Sweden; 2 Department of Computer Science and Informatics, Jönköping University, 551 11 Jönköping, Sweden; 3 Department of Industrial Engineering and Management, University of Gävle, 801 76 Gävle, Sweden

**Keywords:** Decision-making, Fuzzy-AHP, Fuzzy-TOPSIS, Manufacturing relocation, Offshoring, Reshoring

## Abstract

Manufacturing relocation decisions are complex because they involve combinations of location modes like offshoring or reshoring, and governance modes like insourcing or outsourcing. Furthermore, the uncertainty involved in the decision-making process makes it challenging to reach a right-shoring decision. This study presents a hybrid fuzzy-AHP-TOPSIS model to support generic relocation decisions. Industry experts were involved in a pairwise comparison of the competitive priorities’ decision criteria. A meta-synthesis of empirical studies is used to generate theoretical relocation scenarios. The presented hybrid model is used to rank the relocation scenarios in order to identify the most pertinent alternative. The resiliency of the solution is presented through a sensitivity analysis. The results indicate that the proposed hybrid model can simultaneously handle all the main relocation options involving governance modes. Based on the input data in this study, the competitive priorities criteria quality, time and cost are shown to have a strong impact, whereas the sustainability criterion has a weak impact on the choice of relocation option. The research presented in this paper contributes to the research field of manufacturing relocation by demonstrating the suitability of the hybrid fuzzy-AHP-TOPSIS model for relocation decisions and the resilience of the results. Furthermore, the research contributes to practice by providing managers with a generic relocation decision-support model that is capable of simultaneously handling and evaluating various relocation alternatives.

## Introduction

Manufacturing relocations such as offshoring and reshoring, involving governance modes such as insourcing and outsourcing, have gained considerable interest among researchers, practitioners and policymakers (Fratocchi et al. 2016; Hilletofth et al. 2019a; Ketokivi et al. 2017). Offshoring or relocation of the first degree (Barbieri et al. 2019) has been sustained by the idea of having a cost advantage by locating low-value manufacturing activities globally to support local operations (Lewin and Peeters 2006). However, this strategy has been criticized due to inherent problems related to poor quality, loss of flexibility or too long lead times (Kinkel and Maloca 2009). Furthermore, the initial offshoring decisions were often myopic as they were based mainly on cost advantage (Eriksson et al. 2018) and lacked a holistic perspective that consider other advantages, such as responsiveness (Gray et al. 2017). More recently, the COVID-19 pandemic has reiterated the need to build up resiliency and responsiveness across global supply chains (Strange 2020). The need to increase responsiveness has motivated manufacturers to consider different relocations of the second degree, one of which is reshoring to the home country (Barbieri et al. 2019). In this study, reshoring is defined as the relocation of manufacturing back to the home country (Gray et al. 2013). Furthermore, the scope of relocations comprises a location change of either a component, product, process, factory or supplier. While there is consensus that the offshoring decision-making process need to be managed effectively (Ishizaka et al. 2019), the same challenge is observed in the reshoring decision-making process because more decision criteria than mere cost are considered (Boffelli et al. 2020). Nonetheless, in both offshoring and reshoring, the issue of decision support has been inadequately addressed by the research community.

The lack of support for relocation decisions is a consequence of the complexity of the relocation decision-making process due to multiple decision criteria, relocation alternatives and decision makers involved. Furthermore, governance modes, such as insourcing and outsourcing, need to be considered in relocations (Bals et al. 2016; Mugurusi and de Boer 2013). Offshoring and outsourcing are often addressed together. A recent special issue has focused on developing offshoring and outsourcing decision support (Ishizaka et al. 2019). The highlights of the special issue are the advanced decision-support models for offshoring and outsourcing, and the need for fuzzy logic-based models to handle vagueness and uncertainty (Ishizaka et al. 2019). Reshoring and insourcing are also often addressed together (Bals et al. 2016; Foerstl et al. 2016). Both offshoring and reshoring are complex as these involve more than just a one-way location choice (Gray et al. 2013; Mugurusi and de Boer 2013). Rather, these decisions involve multidimensional factors, both qualitative and quantitative, that need to be considered in a constantly changing manufacturing landscape, as well as the associated uncertainty (Kaur et al. 2019; Tate et al. 2014). For instance, the disruption of supply chains caused by the COVID-19 pandemic, is expected to increase reshoring to the home country (Barbieri et al. 2020). To bypass the economic losses caused by these uncertainties, a proactive approach to relocation decision-making is necessary for the future. To aid practitioners in accomplishing this, the development of manufacturing relocation decision-support models is timely and relevant. Moreover, as more companies turn towards digitalizing their manufacturing processes, a digitalized form of decision support is desirable (e.g., Baryannis et al. 2019; Kaur et al. 2019).

Dynamic models for supporting complex offshoring and reshoring decision-making processes have been developed and implemented in the past. For example, the viable system model for offshoring claims that companies consist of at least five subsystems (i.e., operations, coordination, resource utilization, opportunity and threat detection, and top management) that are dynamically adaptive to one another; furthermore, the success of offshoring depends on how quickly these subsystems are able to cope with internal and external variances (Mugurusi and de Boer 2013). While developing complex models for offshoring decisions, several challenges have been identified, and the need for simpler heuristics has been advocated (Kaur et al. 2019). Therefore, it is paramount that a behavioural approach be adopted to address the relocation decision-making problem (Mugurusi and de Boer 2013). The behavioural approach has been considered while developing the system dynamics model for reshoring, which indicates that companies are not entirely rational in their decision-making (Boffelli et al. 2020; Gray et al. 2017). As a result, two common features that can be identified in relocation decision-making are vagueness and uncertainty behind these decisions. The vagueness and uncertainty can be handled in most situations, with the help of a mathematical method known as the fuzzy set theory (De Felice et al. 2021; Hilletofth et al. 2019b, 2021).

When faced with a relocation decision, companies must choose among several alternatives based on the two dimensions, commonly referred to as location and governance (Gray et al. 2013), which are interconnected (Bals et al. 2016). Therefore, a decision-making model should be able to consider the different relocation alternatives in these two dimensions. The relocation alternatives are compared based on certain decision criteria. Certain criteria are more important for either offshoring or reshoring (Di Mauro et al. 2018). The goal of a relocation decision is to select the suitable “right-shoring” alternative – one that addresses a company’s manufacturing needs and is resilient over time. The decision on selecting the best right-shoring alternative among a set of options can be made through multi-criteria decision-making (MCDM) models (Rao 2013). Several types of MCDM models have been developed and applied to the manufacturing relocation domain. Hence, an MCDM model is often the best option to understand or predict the final decision (Bertrand and Fransoo 2002).

Several existing MCDM models are promising for certain types of relocation decisions, for example, AHP (Sequeira et al. 2021), DEMATEL (Chakraborty et al. 2018), ELECTRE (Ray et al. 2015), TOPSIS (Xu et al. 2017), VIKOR (Huang et al. 2020), among others. Thus, it becomes crucial to choose the MCDM best suited for the application at hand. In manufacturing relocation decisions, complexity, vagueness and uncertainty are inherent challenges. The MCDM model for such a decision situation should be able to handle these challenges. Considering the complexity in making decisions, the trade-offs between decision criteria can be managed by assigning weights to the respective decision criteria. AHP includes several features that make it an appropriate model, and its usefulness has been demonstrated for the initial screening purpose of reshoring decisions (Sequeira et al. 2021). Next, considering the uncertainty in making decisions, fuzzy logic-based models are appropriate because they incorporate the mathematical concept of fuzzy sets to cope with the uncertainties. Such models are useful in group decision-making situations, where several management executives are charged with making the final relocation decision (Hilletofth et al. 2021; Sequeira et al. 2021). When too many alternatives are available in a decision-making situation, TOPSIS could be considered an appropriate MCDM model. Furthermore, the TOPSIS method calculates the distance between the most and the least ideal solutions, something that practitioners find easier to understand than other MCDM methods (Arunyanart et al. 2021). Therefore, a fuzzy set-based model, such as fuzzy-AHP, becomes highly suitable for calculating the weights of the criteria considered during a relocation decision (De Felice et al. 2021). To identify the best relocation alternative, fuzzy-TOPSIS is an appropriate option (e.g., Arunyanart et al. 2021). A manufacturing relocation decision relies on the availability of relevant criteria, as well as the possibility to identify the best alternative. Hence, the goal of this study is to develop a hybrid model that includes both fuzzy-AHP and fuzzy-TOPSIS and demonstrate how it can be used to evaluate alternative relocation options that might include both location and governance issues.

The remainder of this paper is organized as follows. In Sect. 2, a review of manufacturing relocation decisions and fuzzy-MCDMs is presented. Section 3 presents a model to generate relocation scenarios, calculate the importance of the decision criteria and ranking of the generated scenarios. In Sect. 4, the numerical results, and sensitivity analysis are presented. Section 5 discusses the results and the model, while Sect. 6 concludes the paper.

## Literature review

The two research fields that are relevant to the relocation decisions in this study are reviewed, namely manufacturing relocation decisions and fuzzy-MCDM models. First, the manufacturing relocation decision field is presented. Its focus is on the relocation direction, for example, reshoring an outsourced activity to an external supplier or reshoring for insourcing to the home installations of an outsourced activity. Second, the fuzzy-MCDM field is briefly presented, with a focus on the two fuzzy decision-making methods used in this study – fuzzy-AHP and fuzzy-TOPSIS.

### Manufacturing relocation decision-making frameworks

The manufacturing offshoring decision-making process has received attention in the literature. A review of offshoring argues that decision-makers have to specify at least three fields, which are interconnected: (1) the value chain activity, (2) the location, and (3) governance mode. Furthermore, both location and governance modes need to be continuously evaluated (Schmeisser 2013). Another review on the offshoring decision-making process assesses 25 years’ worth of research in this area and develops an integrative framework of this process (Mihalache and Mihalache 2016). The framework supports a holistic view on the offshoring decision; however, it does not detail the stages involved in the decision-making process. A recent review on the offshoring decision-making process has been conducted on the same topic, with the importance of MCDM decision-support models in the process (Pereira et al. 2019). However, it does not detail the various stages involved in the process. In all the above-mentioned reviews, the scope of offshored activities has been wider than just the manufacturing function. In the recent review, an example is the AHP model that is used to evaluate supply chain risks in offshoring and outsourcing decisions, and choose the best option among five alternatives (Schoenherr et al. 2008). While the importance of similar decision-support models has been articulated in offshoring and outsourcing decisions, AHP, TOPSIS and fuzzy-MCDMs have been applied to the offshoring and outsourcing decision-making process (Ishizaka et al. 2019).

**Fig. 1:**
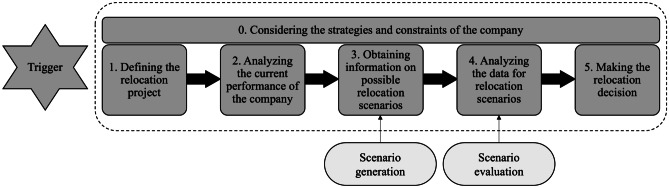
Manufacturing relocation decision-making process (based on Bals et al. 2016; Boffelli et al. 2018; Hilletofth et al. 2021)

The manufacturing reshoring decision-making process, including their governance modes, can be visualized through some frameworks, of which six have been mentioned in the reshoring literature (Bals et al. 2016; Benstead et al. 2017; Boffelli et al. 2018, 2020; Boffelli and Johansson 2020; Hilletofth et al. 2021; Joubioux and Vanpoucke 2016). In the first framework, the decision-making process consists of an evaluation of the push/pull factors and benefits of relocation in order to choose one of the three relocation alternatives: further offshoring, continue offshoring or reshoring (Joubioux and Vanpoucke 2016). However, the framework excludes the governance modes in the relocation decision. The second framework acknowledges the interconnectedness of location decisions and governance modes; as a result, in total, nine unique alternatives for reshoring and insourcing are observed in practice (Bals et al. 2016). It has been added that the reshoring and insourcing decision-making process comprises five generic steps (Bals et al. 2016), which are also pictured in Fig. 1. This decision-making process has been further explored; as a result, the concept of *trigger* (Benstead et al. 2017), lead to the third framework (Boffelli et al. 2018, 2020). According to Benstead et al. (2017), contingency factors and implementation issues should be integrated into a holistic set of drivers. However, the focus is on the content of the reshoring drivers rather than on a decision support model for practitioners. Subsequently, a fourth framework has been developed for both offshoring and reshoring decisions, proposing to move beyond the notion of drivers and barriers and use a generic term for factors (Boffelli and Johansson 2020). In the most recent and the fifth framework, the reshoring decision-making process is tailored to evaluate reshoring decisions and is further accompanied by several types of decision-support tools (Hilletofth et al. 2021).

To identify a relocation, the executive board, who is in charge of relocation decisions, needs to understand the current corporate strategies and constraints of the manufacturing company (i.e., Step 0) (Gray et al. 2017). The executive board then needs to define the relocation project (i.e., Step 1) (Hilletofth et al. 2021). Next, analysts should gather data and analyze the current corporate performance and compare it with the desired performance. During this process (Step 2), the company is required to gather relevant information about the cost and the investment required, existing and required production capacities, machinery selection or the current state of a new product development process, among others (Boffelli et al. 2020). After gathering this information, the executive board assembles information on possible relocation alternatives (Step 3), for example, outsourcing to a local supplier, relocating to a different region in the home country or relocating to the headquarters in the home country. Thereafter, the analysts analyzes the data for the different reshoring alternatives (Step 4) before arriving at the last step of the relocation process, where a final alternative is presented (Step 5). Several types of decision-support models are required to aid in the various steps of the process (Hilletofth et al. 2021). In this paper, the steps to generate and evaluate scenarios are described and applied in the hybrid fuzzy-AHP-TOPSIS model.

**Fig. 2:**
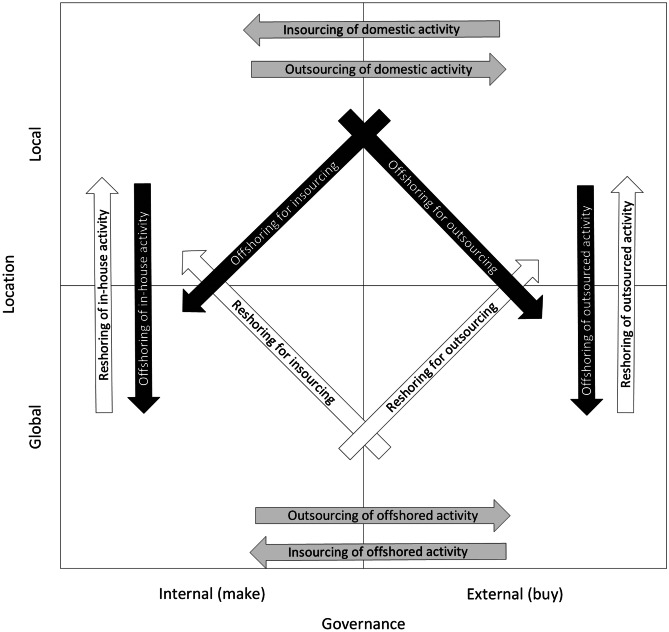
Relocation decisions (based on Eriksson et al. 2021 and Gray et al. 2013)

When deciding on how to supply a market, two main decision types must be considered: (1) location decision (i.e., local or global) and (2) governance decision (i.e., make or buy). Both types are argued to be interconnected (Bals et al. 2016; Eriksson et al. 2021; Gray et al. 2013). Based on the two decision types, there can be four combinations to supply a market (Fig. 2): (1) a manufacturer’s own production in the local market (Quadrant 1), (2) production by suppliers in the local market (Quadrant 2), (3) a manufacturer’s own production in the global market (Quadrant 3) and (4) production by suppliers in the global market (Quadrant 4). Twelve types of manufacturing relocations can thus be distinguished, of which four are linked to offshoring (black arrows) and four are linked to reshoring (white arrows). The remaining four are pure governance decisions (grey arrows). It is acknowledged that more types of decisions could be added, such as a partner selection decision or a coordination decision (Mihalache and Mihalache 2016), which would generate more alternatives. Although nearshoring decisions are not considered in this study, it is possible to have a regional dimension to incorporate such decisions (Bals et al. 2016). However, adding this dimension would increase the number of alternatives.

The different relocation types are driven by various factors. For offshoring and outsourcing options, cost reduction is viewed as a major factor, while for reshoring and insourcing options, value creation is an important factor (Di Mauro et al. 2018; Fratocchi et al. 2016; Johansson et al. 2019). The value creation is expressed through improvements in quality, time, innovation, flexibility and sustainability factors (Kandil et al. 2020). However, for offshoring and outsourcing, quality is affected differently, depending on the governance modes for offshoring (Bruccoleri et al. 2019). For reshoring decisions, it has been observed that cost is usually not the most important factor when moving production back to a high-cost country; instead, quality and flexibility are usually the most significant factors (Dachs et al. 2019; Di Mauro et al. 2018). Sustainability deserves more attention (Orzes and Sarkis 2019), except for a few studies indicating that improved environmental and social pillars of sustainability play a role in reshoring (Ashby 2016). For insourcing decisions, innovation is found to be a crucial factor because insourcing can increase co-location benefits between R&D and production (Lica et al. 2020). However, if co-location has little influence on innovation, then outsourcing is preferable (Mol and Kotabe 2011). As can be observed, a large number of different aspects need to be considered in a relocation decision, whether it includes outsourcing, insourcing, offshoring or reshoring, or sometimes a combination of some of these. This complexity in relocation decisions can be handled by the hybrid fuzzy-AHP-TOPSIS model as demonstrated further on.

### Fuzzy-MCDM

To approach the decision-making domain, many different methods have been proposed over the years. One way of grouping these methods is known as MCDM, and some of the most common MCDM models have been presented in the introduction. However, these models do not explicitly consider the uncertainty aspects of decisions, for example, different opinions among decision makers or the fluctuation in the values of the decision criteria. To incorporate uncertainties in decision-making, the fuzzy set theory has been used, together with the existing MCDM models. As a result of this union, several different models have been proposed and applied to different decision-making domains, including AHP for manufacturing relocations, such as offshoring (De Felice et al. 2021; Schoenherr et al. 2008) and reshoring (Sequeira et al. 2021), while TOPSIS has only been applied to offshoring (Kaur et al. 2019). Two of the fuzzy-MCDM models that have been applied to the manufacturing relocation domain are presented in more detail in the following subsections, namely fuzzy-AHP and fuzzy-TOPSIS.

#### Fuzzy-AHP

Fuzzy-AHP is the second most used MCDM model after traditional AHP (Ho 2008; Liu et al. 2020; Mardani et al. 2015). The reason for implementing fuzzy-AHP instead of AHP is that the natural numbers in AHP cannot entirely express qualitative comparisons. During criteria comparison, fuzzy sets are used instead of crisp sets, as shown in Table 1.

**Table 1:** Scale of preference between two criteria in fuzzy-AHP (Saaty 1980)

**Fuzzy set**	**Verbal fuzzy judgment**
(1,1,1)	Equal preference
(1,3,5)	Moderate preference
(3,5,7)	Strong preference
(5,7,9)	Very strong preference
(9,9,9)	Extremely strong preference

Fuzzy sets are used to express the experts’ preferences. For example, if criterion X moderately dominates criterion Y, then the fuzzy set (1, 3, 5), which represents suitable fuzziness, is entered in the row corresponding to X and the column corresponding to Y. Furthermore, the reciprocal is entered in the row corresponding to Y and the column corresponding to X (Routroy and Pradhan 2013). When such a pairwise comparison has been done for all the decision criteria, a pairwise matrix (A) is obtained, which is represented in Eq. (1). Next, an extended analysis is used for the fuzzy-AHP, according to the procedure suggested by Chang (1996). Accordingly, the extended analysis values and degrees of possibility are calculated for each decision criterion. When done, these values are normalized to obtain final weights (Sequeira et al. 2021).

1 $$\mathrm A=\begin{pmatrix}{\mathrm a}_{11}&\cdots&{\mathrm a}_{1\mathrm n}\\\vdots&\ddots&\vdots\\{\mathrm a}_{\mathrm n1}&\cdots&{\mathrm a}_{\mathrm{nn}}\end{pmatrix},\;and\;\mathrm a\;_{\mathrm{ij}}=1\;\mathrm{if}\;\;i=\mathrm j$$

Fuzzy-AHP has been explored in manufacturing relocation decisions. In one study, three overall criteria from the operations capabilities literature were used in fuzzy-AHP to reach a reshoring decision (Pal et al. 2018). The reshoring decision consisted of four alternatives: make-local, make-nearshore, buy-local and buy-nearshore. The study considered nearshoring as one of the alternatives of reshoring, where the manufacturing was returned close to the home country. In another study, six criteria from the overall competitive priorities, which have been described in the literature, were applied to an industrial setting to evaluate whether or not the reshoring process should be carried out (Sequeira et al. 2021). The study proposed that the fuzzy-AHP method would be suitable for the initial screening of reshoring decisions and recommended its use to determine whether a practitioner should either continue or exit the reshoring decision-making process before taking any costly action. In another study, eight criteria, based on the factors suggested by Ellram (2013), were used with fuzzy-AHP to rank their importance and perceived risks (White and Borchers 2016). However, the study did not delve into developing any relocation alternatives.

The studies that address offshoring decisions combine both location decisions and governance modes. One study applied 19 overall criteria in a joint fuzzy-AHP and fuzzy-TOPSIS method to model combined offshoring–outsourcing decisions (Kaur et al. 2019). The goal was to rank eight suppliers. Another study took a sustainability approach to make joint offshoring–outsourcing decisions as such decisions are often associated with weak and poorly enforced environmental regulations (Awasthi et al. 2018). The study considered six overall criteria that also encompassed the sustainability aspect of potential sub-suppliers.

#### Fuzzy-TOPSIS

Fuzzy-TOPSIS is a practical and useful model for ranking and selecting among several possible alternatives by measuring Euclidean distances that represent proximity to alternatives. The advantage of using fuzzy-TOPSIS is its ability to apply fuzzy sets in order to express the relative importance of the applied criteria. The procedure for fuzzy-TOPSIS has been well established in several applications (e.g., Co 2017; Govindaraju et al. 2015). This subsection briefly presents the fuzzy-TOPSIS model and the process to calculate relocation decision recommendations. It describes the nine different steps in the fuzzy-TOPSIS process.

In the first step, ratings are assigned to the criteria. Assume that *n* criteria are involved in the decision. Each criterion is evaluated against *m* possible alternatives. In the second step, the fuzzy ratings for each criterion are calculated, which are described as triangular fuzzy numbers ${\tilde{d }}_{ij}=({a}_{ij},{b}_{ij}, {c}_{ij})$. In the third step, a fuzzy decision matrix is created. The fuzzy decision matrix ($\tilde{D }$) for *n* criteria and *m* alternatives is constructed and described in Eq. (2):

2 $$\tilde{D }=\left[\begin{array}{cccc}{\tilde{d }}_{11}& {\tilde{d }}_{12}& \dots & {\tilde{d }}_{1n}\\ {\tilde{d }}_{21}& {\tilde{d }}_{22}& \dots & {\tilde{d }}_{2n}\\ \dots & \dots & \dots & \dots \\ {\tilde{d }}_{m1}& {\tilde{d }}_{m2}& \dots & {\tilde{d }}_{mn}\end{array}\right]$$

In the fourth step, the fuzzy ratings are normalized using a linear scale transformation. In this step, the standard TOPSIS method has been modified in this study by using relative linguistic labels (e.g., very poor, poor, neutral, good, very good). Relative linguistic labels are preferred because their use allows improved communication among different stakeholders in a relocation decision situation (Hilletofth et al. 2019b), and it supports the intuitive decision-making that can be observed in relocation decisions (Boffelli et al. 2020). For instance, the label *good* for a criterion (cost or quality) means that the relocation decision has a positive impact on the criterion (decrease in cost or increase in quality). A consequence of using relative linguistic labels is the simplification of the fuzzy-TOPSIS process since all criteria can be considered benefit-type ones. The normalized fuzzy decision matrix $\stackrel{\sim }{{{R}}}$ is given in Eqs. (3) and (4):

3 $$\tilde{R }={[\tilde{r }ij]}_{mxn}, i=1, 2, \dots m;j=1, 2, \dots , n$$

4 $${\widetilde r}_{ij}=\left(\frac{a_{ij}}{c_j^\ast},\frac{b_{ij}}{c_j^\ast},\frac{c_{ij}}{c_j^\ast}\right)\;and\;c_j^\ast=\max_i\{c_{ij}\}$$

In the fifth step, the weighted normalized matrix is calculated by a scalar multiplication with the weights obtained in the fuzzy-AHP and is given by ${\stackrel{\sim }{{{v}}}}_{{{i}}{{j}}}=\left({\stackrel{\sim }{{{r}}}}_{{{i}}{{j}}}\right)\cdot({{{w}}}_{{{i}}})$. In the sixth step, the ideal positive (${{{A}}}^{\boldsymbol{*}}$) (Eq. (5) and the ideal negative solutions (${{{A}}}^{-}$) (Eq. (6)) are calculated. In the seventh step, the Euclidean distances of each alternative from the ideal solutions are calculated (${{{d}}}_{{{i}}}^{\boldsymbol{*}}$, ${{{d}}}_{{{i}}}^{-}$) (Eqs. (7) and (8)). In the eighth step, the closeness coefficient (${{{C}}{{C}}}_{{{i}}}$) is calculated (Eq. (9).

5 $$A^\ast=\left(\widetilde v_1^\ast,\widetilde v_2^\ast,\dots,\widetilde v_m^\ast\right)\;\;\;\;\;\;where\;\widetilde v_j^\ast=\underset i{\mathit{max}}\{v_{ij3}\}$$

6 $$A^-=\left(\widetilde{\mathrm v}_1^\ast,\widetilde{\mathrm v}_2^\ast,\dots,\widetilde v_m^\ast\right)\;\;\;\;\;\;\mathrm{where}\;\widetilde v_j^\ast=\max_i\{v_{ij1}\}$$

7 $$d_i^\ast=\sum\limits_{j=1}^nd_v({\widetilde v}_{ij},\widetilde v_j^\ast)$$

8 $${d}_{i}^{-}=\sum\limits_{j=1}^n{d}_{v}({\tilde{v }}_{ij}, {\tilde{v }}_{j}^{-})$$

9 $${cc}_{i}=\frac{{d}_{i}^{-}}{{d}_{i}^{-}+{d}_{i}^{*}}$$

In the ninth and final step, the alternatives are ranked based on the decreasing order of ${CC}_{i}$. The best alternative is the one closest to the fuzzy positive ideal solution (FPIS) and farthest from the fuzzy negative ideal solution (FNIS). As additional assistance to decision makers, using the values of ${CC}_{i}$, the relocation alternatives can be further grouped into five classes (Chen et al. 2006). The classification provides an explanation in natural language to the decision makers (Table 2).

**Table 2:** Decision recommendation (Chen et al. 2006)

**Closeness coefficient**	**Decision recommendation**
${CC}_{i} \in [\mathrm{0,0.2})$	Do not evaluate
${CC}_{i} \in [\mathrm{0.2,0.4})$	Evaluate with high risk
${CC}_{i} \in [\mathrm{0.4,0.6})$	Evaluate with low risk
${CC}_{i} \in [\mathrm{0.6,0.8})$	Evaluate
${CC}_{i} \in [\mathrm{0.8,1}]$	Strongly evaluate

Fuzzy-TOPSIS has been explored in manufacturing relocation decisions. In one study, fuzzy-TOPSIS was used to rank eight suppliers in joint offshoring and outsourcing decisions (Kaur et al. 2019). The rankings from the fuzzy-TOPSIS were also compared with those from other MCDM models. In another study, fuzzy-TOPSIS was explored in reshoring of outsourced activities in the food industry (Tsimiklis and Makatsoris 2019). The fuzzy-TOPSIS model was optimized by considering three suppliers. The order allocation was done proportionally to the weights of the alternatives. In yet another study, fuzzy-TOPSIS integrated with goal programming was used to select the most promising manufacturing project out of 15 projects, to be outsourced to a supplier in Taiwan (Wei et al. 2019). The goal programming technique consisted of multiple objectives, which were then divided into two objectives, namely cost and benefit. The fuzzy-TOPSIS was integrated to overcome the challenges in goal programming.

## Proposed model for generation of scenarios and evaluation of alternatives

**Fig. 3:**
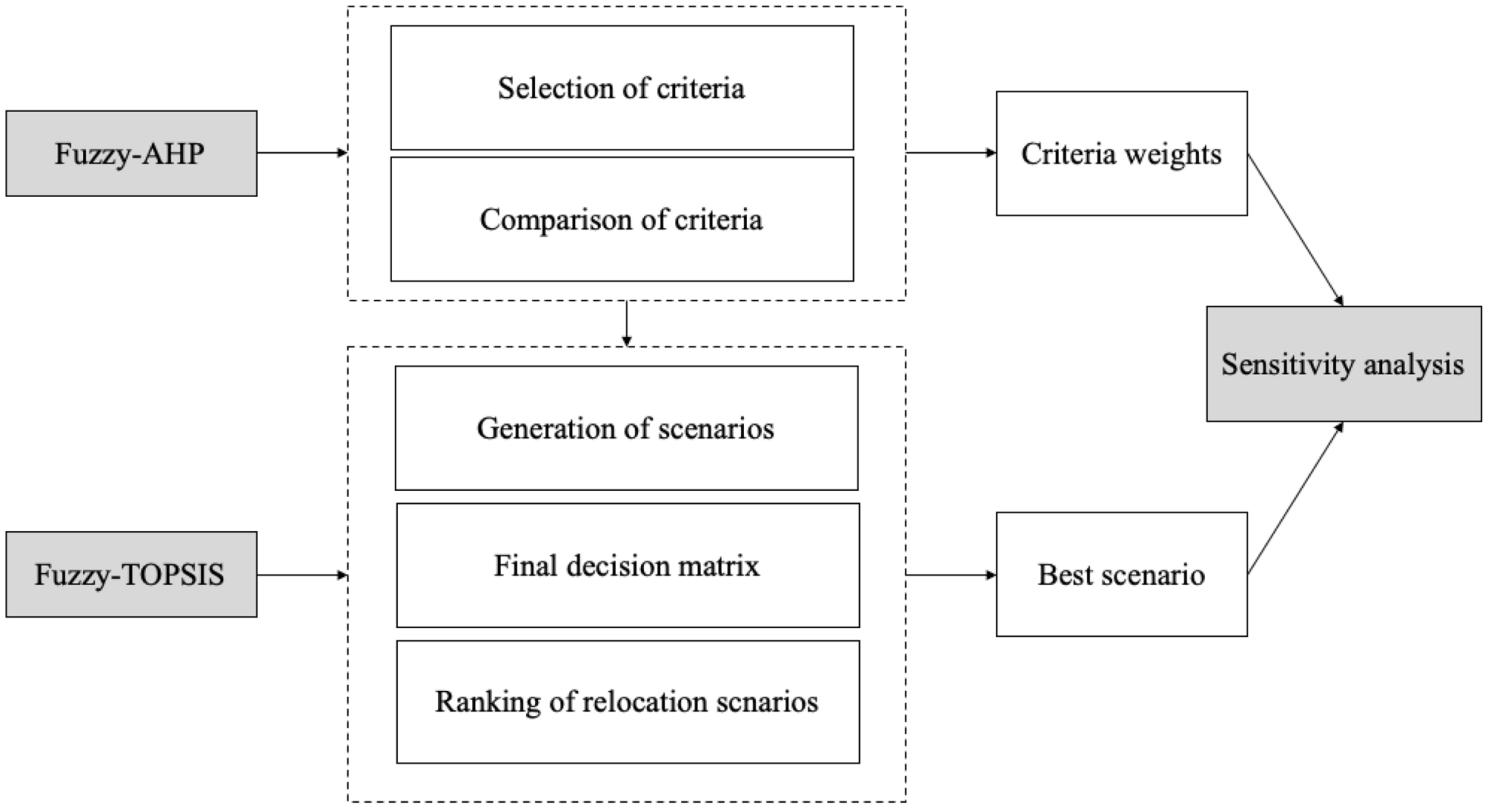
Framework for hybrid fuzzy-AHP-TOPSIS

To develop the hybrid fuzzy-AHP-TOPSIS model, the following framework is proposed (Fig. 3). The goal of the model is to evaluate relocation alternatives out of a given set of options.

Based on the framework, the first step is to select the relevant relocation criteria. The relocation criteria can be considered factors that influence relocation decisions and have been described in the literature when addressing relocation drivers, barriers and enablers (Barbieri et al. 2018; Engström et al. 2018a, b; Stentoft et al. 2016b; Wiesmann et al. 2017). In this paper, six high-level criteria that are related to established competitive priorities have been chosen since the main goal of any relocation decision is to enhance a company’s competitive advantage (Sansone et al. 2020). The six criteria are cost, quality, time, flexibility, innovation and sustainability (Hilletofth et al. 2019b; Sansone et al. 2017). Using competitive priorities is one of the ways to test the model; furthermore, it has been proposed as a relevant group of criteria for relocation decisions (Hilletofth et al. 2021; Sequeira et al. 2021). Other groups of criteria that could be considered are risks, resources, global dynamics and contexts (Benstead et al. 2017; Sequeira et al. 2020).

In the second step, a pairwise comparison of the six criteria was carried out using fuzzy sets, according to the method followed for developing fuzzy logic-based and other fuzzy-MCDM models (Hilletofth et al. 2021; Sequeira et al. 2021). A panel of five industry experts from a large company that manufactures air conditioning and heating combination units, conducted the comparisons individually, when were then averaged. Headquartered in Småland County in Sweden, the company has been active in both offshoring and reshoring relocations between 2015 and 2018. The industry experts belong to the company’s top management team and have expertise in making relocation decisions (for a complete description, see Appendix 1). All the involved experts agreed that the set of six competitive priorities is one of the relevant criteria groups when dealing with relocation decisions. Following the pairwise comparison of the criteria, the priority weight of each criterion was calculated using fuzzy-AHP.

In the third step, relocation scenarios were generated to represent each of the 12 relocation types through a case study review (the results are demonstrated in Table 3). To generate realistic relocation scenarios, case study evidence from the literature was extracted and analyzed by adapting a meta-synthesis technique (Boffelli and Johansson 2020; Hoon 2013). The purpose for using the technique was to find realistic scenarios corresponding to each of the 12 manufacturing relocations, but not to develop theories concerning the background of the relocations. The search for empirical studies was conducted using the Scopus database, with keywords pertaining to manufacturing relocation, including location and governance modes (Appendix 2)and the results were compared with those of other studies (Barbieri et al. 2018; Boffelli and Johansson 2020). One criterion for inclusion was to include articles that contain empirical cases and were published in 2000–2020. One criterion for exclusion was to exclude those cases that do not deal with a relocation decision in the past. Therefore, the articles that propose decision support for future relocations were excluded. Another criterion for exclusion was related to articles with insufficient description of the case or articles that do not specify both location and governance modes. After applying the inclusion and exclusion criteria, the resulting sample consisted of 32 articles (the search process can be found in Appendix 2). The sample comprised multiple case studies that provided descriptions of both offshoring and reshoring cases (e.g., Di Mauro et al. 2018; Engström et al. 2018b), as well as single case studies (e.g., Ashby 2016; Gylling et al. 2015). No case study for offshoring for insourcing was identified. Therefore, the snowballing technique was used to identify two empirical cases from one article (i.e., Bals et al. 2016). As the purpose of using the meta-synthesis was only to find realistic scenarios, the concluding steps such as building theory and discussing the results of meta-synthesis (Hoon, 2013) are not included. Based on the developed relocation scenarios, the hybrid fuzzy-AHP-TOPSIS model could be tested and evaluated.

**Table 3:** Relocation scenarios (VP = very poor; P = poor; N = neutral; G = good; VG = very good)

**Scenario**	**Relocation type**	**Cost**	**Quality**	**Time**	**Flexibility**	**Innovation**	**Sustainability**	**Selected sources**
1	Reshoring of in-house activity	N	G	N	N	N	N	Di Mauro et al. 2018; Engström et al. 2018a
2	Reshoring for insourcing	N	G	N	N	N	N	Di Mauro et al. 2018; Engström et al. 2018a
3	Reshoring of outsourced activity	N	VG	N	N	N	N	Ashby 2016; Gray et al. 2017
4	Reshoring for outsourcing	N	N	VG	N	VG	N	Grandinetti and Tabacco 2015
5	Offshoring for insourcing	N	N	N	VG	VG	N	Bals et al. 2016
6	Offshoring for outsourcing	VG	P	P	N	N	N	Di Mauro et al. 2018; Gylling et al. 2015
7	Offshoring of outsourced activity	P	VP	N	N	N	N	Song et al. 2007
8	Offshoring of in-house activity	VG	N	G	N	N	N	Di Mauro et al. 2018; Engström et al. 2018a
9	Insourcing of domestic activity	G	N	N	VG	N	N	Drauz (2014)
10	Insourcing of offshored activity	G	N	N	G	N	N	Caputo and Palumbo (2005)
11	Outsourcing of domestic activity	N	G	VG	G	N	N	Rehme et al. 2013
12	Outsourcing of offshored activity	G	N	N	N	P	N	Nujen et al. 2018
13	No change	N	N	N	N	N	N	

**Fig. 4:**
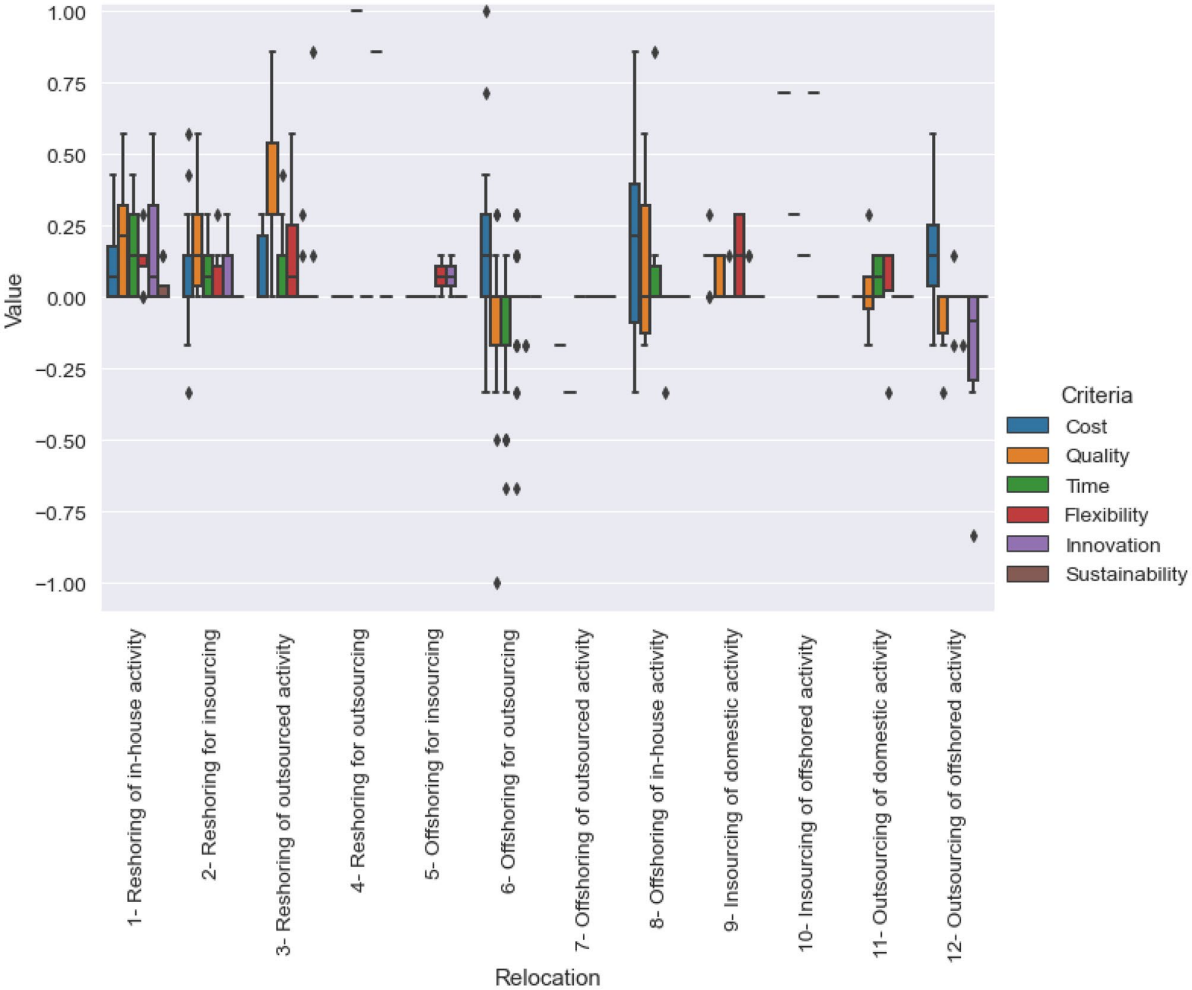
Normalized frequency of criteria mentioned in case studies in each relocation

The unit of analysis in the meta-synthesis was a relocation decision. In total, 115 decisions were identified from the 32 articles (the complete list can be found in Appendix 3). Next, in each article, the parts addressing the decision criteria as drivers or barriers in the case study description, as well as in the findings section of the article, were coded (Boffelli and Johansson 2020). The coding consisted of assigning a value of + 1 or $-$ 1, based on whether the identified relocation criterion was expressed as a positive (driver) or a negative (barrier) impact on the relocation. This way of assigning values to a concept depends on the inherent subjectivity of the texts and is comparable to a sentiment analysis (Mäntylä et al. 2018). In the next step, towards generating the required relocation scenarios, the values were summed up and normalized for each criterion. A boxplot was used to visualize the middle 50% of the values and the median of each criterion for each relocation type (Fig. 4).

For each relocation type, the values were normalized, which allowed the comparison of criteria in a relative sense. Furthermore, the normalization follows a replication logic, meaning that if more sentences are coded for a particular criterion, then it increases the value of that criterion over all the other criteria in that direction. This logic is used to develop theories out of qualitative case studies (Hoon 2013); however, in this study, the intention is not to develop theories but to develop realistic scenarios as input to the model. Finally, the normalized values were transformed to the linguistic labels *very poor* (VP), *poor* (P), *neutral* (N), *good* (G) or *very good* (VG) using a fuzzy scale. The assignment of the 12 relocation scenarios was based on the gathering of scientific evidence related to the relocation domain (Table 3). Finally, during a relocation decision-making process, a scenario (Scenario 13) could be to continue production at the current location, suggesting business as usual.

In the fourth step, the fuzzy decision matrix was obtained, to which the fuzzy-TOPSIS method was applied. In the fifth step, the ${CC}_{i}$ values were computed according to the steps described in the previous section explaining the fuzzy-TOPSIS process. Finally, a sensitivity analysis was carried out to evaluate the resilience (in time) of the results produced by the model. The hybrid fuzzy-AHP-TOPSIS model was carried out using Excel while the sensitivity analysis was performed using Python.

## Numerical results

The results generated by the hybrid fuzzy-AHP-TOPSIS model are presented in this section. Table 4 shows the average values of the five industrial relocation experts’ comparisons of the six relocation criteria with respect to their relative importance in a relocation decision situation from the perspective of a company located in a high-cost country. The experts compared each criterion against every other criterion in a structured, pairwise manner, based on their previous experiences and judgement. The pairwise comparison was based on a nine-point scale, which uses five fuzzy sets, each having lower, middle and upper values. In the first stage, the priority weights of the criteria are calculated using fuzzy-AHP. In Table 4, it can be observed that *quality* has the highest priority weight, indicating that it is the criterion with the highest importance in a relocation decision situation, followed by *time*, *cost* and *flexibility*. The criterion *sustainability* has zero weight (Table 4).

**Table 4:** Average pairwise comparison of criteria among five experts

**Criteria **${{\varvec{C}}}_{{\varvec{i}}}$	**Cost**	**Quality**	**Time**	**Flexibility**	**Innovation**	**Sustainability**	**Final priority weights**
**Cost**	(1,1,1)	(0.18,0.27,0.71)	(0.67,1.51,2.47)	(1.08,2.34,3.8)	(1.46,2.67,3.91)	(3.8,5.8,7.8)	**0.19**
**Quality**	(2.2,4.2,6.2)	(1,1,1)	(3.4,5,6.6)	(3.8,5.8,7.8)	(3.8,5.8,7.8)	(4.2,5.8,7.4)	**0.36**
**Time**	(1.08,1.94,3)	(0.16,0.24,0.56)	(1,1,1)	(2.43,3.64,4.87)	(1.64,3.27,5)	(3.4,5.4,7.4)	**0.22**
**Flexibility**	(0.51,1.38,2.47)	(0.14,0.2,0.39)	(0.89,1.33,1.88)	(1,1,1)	(1.64,2.87,4.2)	(3.8,5,6.2)	**0.15**
**Innovation**	(1.7,2.55,3.54)	(0.14,0.18,0.28)	(0.34,0.82,1.54)	(0.5,0.95,1.54)	(1,1,1)	(2.83,3.64,4.47)	**0.08**
**Sustainability**	(0.14,0.18,0.28)	(0.15,0.2,0.4)	(0.15,0.21,0.42)	(0.31,0.35,0.43)	(0.9,1.33,1.89)	(1,1,1)	**0.00**

According to the subsequent steps of the hybrid model, fuzzy-TOPSIS is applied to the relocation alternatives (Fig. 3). The first step is to assign a fuzzy scale (represented by a triangular fuzzy membership function) to the relative linguistic labels (Table 5). In the next step, each scenario in Table 3 is translated using the fuzzy membership functions, followed by the relative values being replaced by fuzzy sets, resulting in the fuzzy decision matrix (Table 6).

**Table 5:** Translation of relative scale to corresponding fuzzy scale

**J Purchasing and Supply Management**	**J Purchasing and Supply Management**
*“Very poor” (VP)*	(1, 1, 3)
*“Poor” (P)*	(1, 3, 5)
*“Neutral” (N)*	(3, 5, 7)
*“Good” (G)*	(5, 7, 9)
*“Very good” (VG)*	(7, 9, 9)

**Table 6:** Fuzzy decision matrix

**Scenario**	**Relocation type**	**Cost**	**Quality**	**Time**	**Flexibility**	**Innovation**	**Sustainability**
1	Reshoring of in-house activity	(3,5,7)	(5,7,9)	(3,5,7)	(3,5,7)	(3,5,7)	(3,5,7)
2	Reshoring for insourcing	(3,5,7)	(5,7,9)	(3,5,7)	(3,5,7)	(3,5,7)	(3,5,7)
3	Reshoring of outsourced activity	(3,5,7)	(7,9,9)	(3,5,7)	(3,5,7)	(3,5,7)	(3,5,7)
4	Reshoring for outsourcing	(3,5,7)	(3,5,7)	(7,9,9)	(3,5,7)	(7,9,9)	(3,5,7)
5	Offshoring for insourcing	(3,5,7)	(3,5,7)	(3,5,7)	(7,9,9)	(7,9,9)	(3,5,7)
6	Offshoring for outsourcing	(7,9,9)	(1,3,5)	(1,3,5)	(3,5,7)	(3,5,7)	(3,5,7)
7	Offshoring of outsourced activity	(1,3,5)	(1,1,3)	(3,5,7)	(3,5,7)	(3,5,7)	(3,5,7)
8	Offshoring of in-house activity	(7,9,9)	(3,5,7)	(5,7,9)	(3,5,7)	(3,5,7)	(3,5,7)
9	Insourcing of domestic activity	(5,7,9)	(3,5,7)	(3,5,7)	(7,9,9)	(3,5,7)	(3,5,7)
10	Insourcing of offshored activity	(5,7,9)	(3,5,7)	(3,5,7)	(5,7,9)	(3,5,7)	(3,5,7)
11	Outsourcing of domestic activity	(3,5,7)	(5,7,9)	(7,9,9)	(5,7,9)	(3,5,7)	(3,5,7)
12	Outsourcing of offshored activity	(5,7,9)	(3,5,7)	(3,5,7)	(3,5,7)	(1,3,5)	(3,5,7)
13	No change	(3,5,7)	(3,5,7)	(3,5,7)	(3,5,7)	(3,5,7)	(3,5,7)

The fuzzy decision matrix is then normalized, and a scalar value is multiplied with the weights obtained from fuzzy-AHP to produce a weighted normalized decision matrix. Following this step, the distances from ideal positive (${A}^{*}$) and ideal negative (${A}^{-}$) solutions are calculated (Table 7).

**Table 7:** Distances of the alternatives from ideal positive (${A}^{*}$) and ideal negative (${A}^{-}$)

**Scenario**	**Relocation type**		**Cost**	**Quality**	**Time**	**Flexibility**	**Innovation**	**Sustainability**
1	Reshoring of in-house activity	(${A}^{*}$)	0.073	0.065	0.085	0.058	0.031	0.000
		(${A}^{-}$)	0.042	0.217	0.049	0.000	0.018	0.000
2	Reshoring for insourcing	(${A}^{*}$)	0.073	0.065	0.085	0.058	0.031	0.000
		(${A}^{-}$)	0.042	0.217	0.049	0.000	0.018	0.000
3	Reshoring of outsourced activity	(${A}^{*}$)	0.073	0.000	0.085	0.058	0.031	0.000
		(${A}^{-}$)	0.042	0.269	0.049	0.000	0.018	0.000
4	Reshoring for outsourcing	(${A}^{*}$)	0.073	0.139	0.000	0.058	0.000	0.000
		(${A}^{-}$)	0.042	0.139	0.132	0.000	0.048	0.000
5	Offshoring for insourcing	(${A}^{*}$)	0.073	0.139	0.085	0.000	0.000	0.000
		(${A}^{-}$)	0.042	0.139	0.049	0.058	0.048	0.000
6	Offshoring for outsourcing	(${A}^{*}$)	0.000	0.217	0.132	0.058	0.031	0.000
		(${A}^{-}$)	0.114	0.065	0.000	0.000	0.018	0.000
7	Offshoring of outsourced activity	(${A}^{*}$)	0.114	0.269	0.085	0.058	0.031	0.000
		(${A}^{-}$)	0.000	0.000	0.049	0.000	0.018	0.000
8	Offshoring of in-house activity	(${A}^{*}$)	0.000	0.139	0.040	0.058	0.031	0.000
		(${A}^{-}$)	0.114	0.139	0.098	0.000	0.018	0.000
9	Insourcing of domestic activity	(${A}^{*}$)	0.034	0.139	0.085	0.000	0.031	0.000
		(${A}^{-}$)	0.084	0.139	0.049	0.058	0.018	0.000
10	Insourcing of offshored activity	(${A}^{*}$)	0.034	0.139	0.085	0.027	0.031	0.000
		(${A}^{-}$)	0.084	0.139	0.049	0.033	0.018	0.000
11	Outsourcing of domestic activity	(${A}^{*}$)	0.073	0.065	0.000	0.027	0.031	0.000
		(${A}^{-}$)	0.042	0.217	0.132	0.033	0.018	0.000
12	Outsourcing of offshored activity	(${A}^{*}$)	0.034	0.139	0.085	0.058	0.048	0.000
		(${A}^{-}$)	0.084	0.139	0.049	0.000	0.000	0.000
13	No change	(${A}^{*}$)	0.073	0.139	0.085	0.058	0.031	0.000
		(${A}^{-}$)	0.042	0.139	0.049	0.000	0.018	0.000

Next, the distance values are summed row by row to calculate the aggregate distance of each alternative (${d}^{*}$ and ${d}^{-}$). After this, ${CC}_{i}$ is calculated, and an initial decision recommendation is given on a global level (Table 8). The column with the heading Direction refers to the movement between quadrants. The decision recommendations are based on the ranges described in Table 2.

**Table 8:** ${d}^{*}$, ${d}^{-}$, ${CC}_{i}$ and decision recommendation for relocations

**Scenario**	**Relocation type**	**Direction**	${{\varvec{d}}}_{{\varvec{i}}}^{\boldsymbol{*}}$	${{\varvec{d}}}_{{\varvec{i}}}^{-}$	${{\varvec{C}}{\varvec{C}}}_{{\varvec{i}}}$	**Decision recommendation**
1	Reshoring of in-house activity	Q3 → Q1	0.312	0.326	0.511	Evaluate with low risk
2	Reshoring for insourcing	Q4 → Q1	0.312	0.326	0.511	Evaluate with low risk
3	Reshoring of outsourced activity	Q4 → Q2	0.246	0.378	0.606	Evaluate
4	Reshoring for outsourcing	Q3 → Q2	0.269	0.361	0.573	Evaluate with low risk
5	Offshoring for insourcing	Q2 → Q3	0.296	0.336	0.531	Evaluate with low risk
6	Offshoring for outsourcing	Q1 → Q4	0.438	0.197	0.311	Evaluate with high risk
7	Offshoring of outsourced activity	Q2 → Q4	0.557	0.067	0.107	Do not evaluate
8	Offshoring of in-house activity	Q1 → Q3	0.267	0.368	0.580	Evaluate with low risk
9	Insourcing of domestic activity	Q2 → Q1	0.289	0.347	0.546	Evaluate with low risk
10	Insourcing of offshored activity	Q4 → Q3	0.316	0.323	0.506	Evaluate with low risk
11	Outsourcing of domestic activity	Q1 → Q2	0.196	0.442	0.692	Evaluate
12	Outsourcing of offshored activity	Q3 → Q4	0.364	0.272	0.428	Evaluate with low risk
13	No change	-	0.385	0.247	0.391	Evaluate with high risk

For obvious reasons, the 12 (plus one) relocation scenarios do not all appear in a single relocation decision situation. Instead, the number of relocation choices is constrained by the location dimension (i.e., the physical location of the manufacturing facility) and the governance dimension before the relocation analysis can be performed. Hence, based on Fig. 2 that shows the relocation options, the company’s activities can be positioned in one of the four quadrants. This means that four separate relocation contexts are possible and need to be investigated separately.

**Fig. 5:**
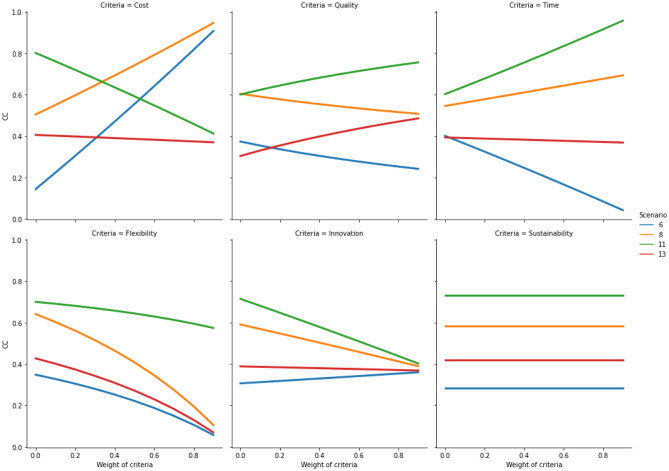
Sensitivity analysis for increasing weights of criteria in quadrant 1

**Fig. 6:**
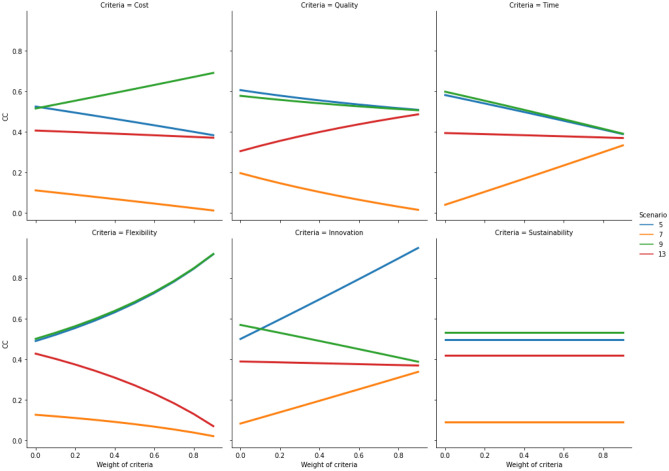
Sensitivity analysis for increasing weights of criteria in quadrant 2

**Fig. 7:**
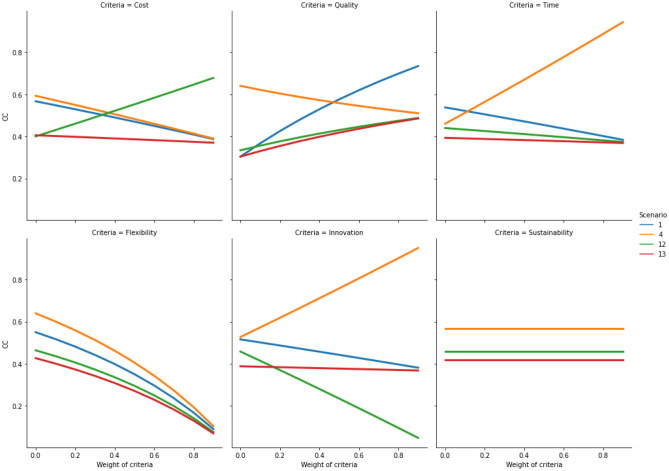
Sensitivity analysis for increasing weights of criteria in quadrant 3

**Fig. 8:**
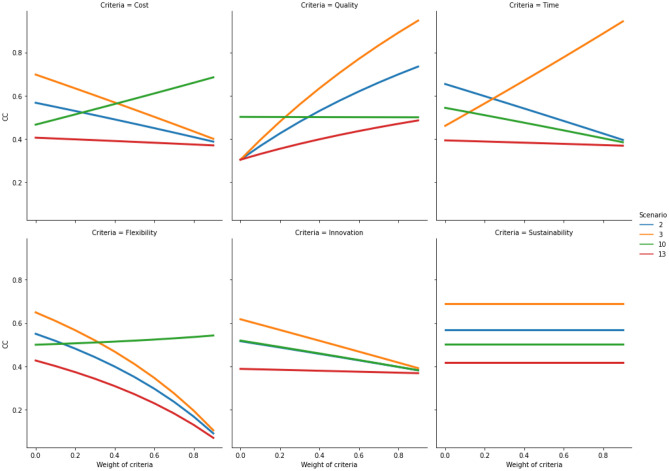
Sensitivity analysis for increasing weights of criteria in quadrant 4

Once the relocation results have been produced, the developed hybrid fuzzy-AHP-TOPSIS model needs to be evaluated in relation to its sensitivity to changes of the criteria weights and the ranking order of the available relocation alternatives. To demonstrate the stability of the results produced by the model, the weight of each of the six criteria is incremented in 10 iterations [0, 0.1, 0.2, 0.3, 0.4, 0.5, 0.6, 0.7, 0.8 and 0.9]. In each iteration, the *CC* value is calculated for all 12 scenarios. The sensitivity results are plotted for cost, quality, time, flexibility, innovation and sustainability in each of the four relocation contexts (i.e., Quadrants 1, 2, 3 and 4). The plots (Figs. 5, 6, 7, and 8) indicate how the *CC* values change for the relocation scenarios, when importance of each criterion is increased in a stepwise manner. As can be observed in the figures in Sects. 4.1, 4.2, 4.3, and 4.4, on some occasions the colored lines, representing different relocation scenarios, cross, indicating a change in the ranking of the relocation scenarios. A company must thus be aware of this behavior. If the evaluation of different relocation scenarios indicates that the lines do not cross for any of the relocation criteria, the correct weight assignment to the different criteria will be of little or no consequence to the ranking, thus indicating a stable situation. But in cases where the lines do cross, it becomes paramount from the company's point-of-view to correctly assign criteria weights.

### Quadrant 1 (Internal production supplying to national market)

If a company’s manufacturing facility is physically situated in a place corresponding to Quadrant 1, the company is faced with four relocation alternatives, as follows: *offshoring for outsourcing*, *offshoring of in-house activity*, *outsourcing of domestic activity* and *no change*. It can be observed that if starting in Quadrant 1, and for the weights obtained in this study, the best decision is to evaluate Scenario 11 (outsourcing of domestic activity; *CC* = 0.692), followed by Scenario 8 (offshoring of in-house activity; *CC* = 0.580), Scenario 13 (no change; *CC* = 0.391) and Scenario 6 (offshoring for outsourcing; *CC* = 0.311).

The sensitivity analysis of the different criteria in Quadrant 1 (Fig. 5) shows that for high importance of the cost criterion, the preference for Scenarios 6 and 8 increases. In contrast, for low importance of the cost criterion, Scenario 11 is preferred. Furthermore, there is limited stability in the ranking of the scenarios for low and middle values of the cost criterion. For the remaining criteria, there is high stability in the ranking of the scenarios. For example, Scenario 11 is the most preferred, followed by Scenarios 8, 13 and 6. For the quality and the time criteria, certain scenarios become preferable than others. For example, Scenario 8 is not preferred for higher values of the quality criterion but is favoured for higher values of the time criterion. For increasing values of the flexibility and the innovation criteria, the ranking order of the scenarios is stable. Furthermore, all scenarios concerning Quadrant 1 become less preferable for increasing values of the flexibility and the innovation criteria. For higher values of the innovation criterion, all scenarios seem to converge towards a point where not one scenario is clearly better than others. The sustainability criterion does not influence the decision in this quadrant, and all scenarios retain their value.

### Quadrant 2 (Outsourced production supplying to national market)

If a company’s manufacturing facility is physically situated in a place corresponding to Quadrant 2, the company is faced with four relocation alternatives, as follows: *offshoring for insourcing*, *offshoring of outsourced activity*, *insourcing of domestic activity* and *no change*. It can be observed that if starting in Quadrant 2 and for the weights obtained in the fuzzy-AHP, the best decision is to evaluate Scenario 9 (insourcing of domestic activity; *CC* = 0.546), followed by Scenario 5 (offshoring for insourcing; *CC* = 0.531) and Scenario 13 (no change; *CC* = 0.391). The model indicates that Scenario 7 (offshoring of outsourcing activity; *CC* = 0.107) is the least preferred option compared with the other relocation alternatives.

The sensitivity analysis of the criteria in Quadrant 2 (Fig. 6) shows stability in the rankings of the scenarios for most of the criteria. Scenarios 9 and 5 are preferred over Scenarios 7 and 13, irrespective of the criteria. Similar to Quadrant 1, there are some differences in how certain criteria make certain scenarios preferable to be evaluated. For example, for increasing values of the cost criterion, it becomes preferable to evaluate Scenario 9, while the other scenarios become less preferable. For increasing values of the quality and the time criteria, Scenarios 9 and 5 are found to be less preferable. For increasing values of the time criterion, all scenarios seem to converge; therefore, for very high values of the time criterion, it does not matter which scenario is selected. In contrast, all scenarios seem to diverge for increasing values of the flexibility criterion. Scenarios 9 and 5 become the most preferable to be evaluated, as opposed to others. For the innovation criterion, all scenarios except Scenario 5, seem to converge. However, it is preferable to evaluate Scenario 5 for increasing values of the innovation criterion. The sustainability criterion does not affect the decision in this quadrant, as all scenarios retain their value.

### Quadrant 3 (Internal production supplying to global market)

If a company’s manufacturing facility is physically situated in a place corresponding to Quadrant 3, the company is faced with four relocation alternatives, as follows: *reshoring of in-house activity*, *reshoring for outsourcing*, *outsourcing of offshored activity* and *no change*. It can be observed that if starting in Quadrant 3 and for the weights obtained this study, the best decision is to evaluate Scenario 4 (reshoring for outsourcing; *CC* = 0.573), followed by Scenario 1 (reshoring of in-house activity; *CC* = 0.511), Scenario 12 (outsourcing of offshored activity; *CC* = 0.428) and Scenario 13 (no change; *CC* = 0.391).

The sensitivity analysis of the criteria in Quadrant 3 (Fig. 7) indicates limited stability in the ranking order, and it depends on the selected criterion. For example, only the scenarios sensitive to the flexibility and the sustainability criteria show stability; meanwhile, there is limited stability in the ranking order of the scenarios sensitive to the other criteria. On one hand, for increasing values of the cost criterion, the preference for Scenario 12 increases, whereas the preference for Scenarios 1 and 4 decreases. On the other hand, for higher values of the quality criterion, the preference for Scenario 1 increases. For the time criterion, all the concerned scenarios, except Scenario 4, become less preferable to be evaluated and converge at high values. Regarding higher values of the flexibility criterion, all scenarios become less preferable to be evaluated. For higher values of the innovation criterion, Scenario 4 is the most preferable to be evaluated, while the sustainability criterion has no impact on the decision in this quadrant, as all scenarios retain their value.

### Quadrant 4 (Outsourced production supplying to global market)

If a company’s manufacturing facility is physically situated in a place corresponding to Quadrant 4, the company is faced with four relocation alternatives, as follows: *reshoring for insourcing*, *reshoring of outsourced activity*, *insourcing of offshored activity* and *no change*. It can be observed that if starting in Quadrant 4 and for the weights obtained in this study, the best decision is to evaluate Scenario 3 (reshoring of outsourced activity; *CC* = 0.606), followed by Scenario 2 (reshoring for insourcing; *CC* = 0.511), Scenario 10 (insourcing of offshored activity; *CC* = 0.506) and Scenario 13 (no change; *CC* = 0.391).

The sensitivity analysis of the criteria in Quadrant 4 (Fig. 8) shows limited stability in the ranking order of the concerned scenarios, and it depends on the selected criterion. For example, in the case of the innovation and the sustainability criteria, there is stability in the rankings, but for the other criteria, the stability is limited to a certain range. For higher values of the cost criterion, Scenario 10 is preferred, while for lower values, Scenario 3 is favoured. For high values of the quality and the time criteria, Scenario 3 is preferred. For high values of the time criteria, the other scenarios seem to converge toward a point. Similar behaviour is observed for the flexibility and the innovation criteria. For the flexibility criterion, all scenarios, except Scenario 10, appear to converge, while for the innovation criterion, all scenarios converge. For high values of the innovation criterion, Scenario 3 is the most preferred, while the sustainability criterion has no impact on the decision in this quadrant, as all scenarios retain their value.

## Discussion

Decision-making in the manufacturing relocation domain has been a high-priority avenue for research; nevertheless, there is a lack of decision-support models covering all available relocation directions (Barbieri et al. 2018; Hilletofth et al. 2019a). The existing frameworks and decision-support models that have been reported in the literature have focused on either offshoring/outsourcing or reshoring/insourcing perspectives, despite growing interest in combinations of both location and governance modes (Gray et al. 2013; Hilletofth et al. 2019a; Ketokivi et al. 2017). In some cases, relocation is perceived as a movement only in the location dimension, without considering governance options, despite both being interconnected. Therefore, relocation decisions deserve a broader picture rather than the existing binary perspective. Moreover, the prevailing COVID-19 pandemic has raised important questions about optimizing a few decision criteria (e.g., cost) in relocation decisions, rather than developing overall resilience in global supply chains. In this paper, location and governance decisions are viewed as interconnected, as previously proposed (Bals et al. 2016). Therefore, this paper addresses these issues by offering a broader picture of both types of relocation and developing a decision-support model that can be used for any type of relocation decisions. Several types of decision support can be involved in a relocation decision-making process, and one type of decision support is a relocation scenario-generation and evaluation model (Hilletofth et al. 2021). Not all the available 13 relocation options are relevant for a company to pursue in a given situation. Each quadrant is associated with at least four relocation options, including a no change option. This allows decision makers to take a holistic perspective on relocation decisions and evaluate all realistic scenarios. Relocation-scenario generation and evaluation require decision makers to explore the different relocation scenarios at hand, which depend on the quadrant in which the manufacturing facility is located.

The relocation scenarios were generated based on a case study description of how (positive or negative) the decision criteria were perceived. A meta-synthesis of 115 case studies published in 32 articles in the relocation literature was carried out, covering all the main types of relocations and governance modes (Appendix 2). Some of the relocations were more common than others. For example, Relocation 6 (offshoring for outsourcing) generated the highest amount of data points in the sample. Thus, this type of relocation was the most common. In contrast, Relocation 4 (reshoring for outsourcing) and Relocation 5 (offshoring for insourcing) generated the lowest amount of data points due to the paucity of the relevant case studies found in the sample. Therefore, these types of relocations were the least common. These relocations still need further investigation in the future through case studies, despite some previous efforts in this direction (Bals et al. 2016). Previous studies on meta-synthesis have examined the reshoring field in order to develop theories pertaining to reshoring (Boffelli and Johansson 2020). In contrast, this meta-synthesis takes a broader view of relocation by including different types of changes in location and governance decisions. The purpose for using the meta-synthesis technique was to find realistic scenarios corresponding to each of the 12 manufacturing relocations, but not to develop theories concerning the background of the relocations. Nevertheless, the technique provided some insights regarding the relocations and the decision criteria. First, not all types of relocations were seen to improve the decision criteria, as some types of relocations were found to be more common than others (Fig. 4). Second, some types of relocations influence the decision criteria in both positive and negative directions (e.g., 2, 6, 8, 11, 12), as data points were found on both positive and negative sides of the box plot (Fig. 4).

Once the relocation scenarios were in place, the hybrid fuzzy-AHP-TOPSIS model was used to evaluate and rank them. The goal of fuzzy-AHP is to rank the criteria that involve uncertainty when conducting a pairwise comparison of the involved relocation criteria. The fuzzy-AHP process follows the reasoning of the involved industry experts (Appendix 1) with respect to the decision criteria. Quality and time are shown to be the most important criteria, while sustainability is the least important criterion. Previous studies have pointed out the issue of the sustainability criterion converging to zero when all the other criteria are more important (Sequeira et al. 2021). The developed model shows the context of a large Swedish company that manufactures air conditioning and heating combination units. The model needs to be adapted while applying to other contexts. Previous models have shown similar results regarding the most and the least important criteria in the context of Swedish transportation manufacturing industry (Sequeira et al. 2021). The demonstrated importance of the criteria is in line with the findings of previous research on relocation in the context of high-cost countries (Di Mauro et al. 2018; Johansson and Olhager 2018). Therefore, the impacts of quality and time on the results are noticeable in the hybrid fuzzy-AHP-TOPSIS model. However, the relocation literature has highlighted the need to understand the sustainability issues (Orzes and Sarkis 2019), despite their regulation in western countries (Pal et al. 2018). The developed model requires to be adapted to the context of the company, and to the external environment that affects the company, for instance, increasing emphasis of the sustainability criterion.

Several existing frameworks cluster relocation criteria under either homogeneous groups (Fratocchi et al. 2016; Wiesmann et al. 2017) or theoretically driven frameworks (Ancarani et al. 2015). Furthermore, existing MCDM models that focus on relocations have applied a holistic set of criteria that encompasses a variety of cost factors (Wei et al. 2019), quality and flexibility (Kaur et al. 2019) and relational factors (Pal et al. 2018). The sustainability factors comprise one group of factors that is argued to be less focused on by MCDM models (Awasthi et al. 2018). In this paper, the chosen set of criteria adopts a perspective on competitive priorities (Hilletofth et al. 2019b; Sansone et al. 2017, 2020) that encompass the factors considered in previous MCDM models (Sequeira et al. 2021). These criteria have been selected for the relocation support model because they represent a holistic way for companies to stay competitive in general and continue to operate in high-cost countries in particular (Sansone et al. 2020). To remain competitive, a company needs to focus on one or more of these criteria. Shortfall in performance has been one of the most common triggers for relocation decisions (Gray et al. 2017). Therefore, and in any relocation decision, these criteria should be evaluated continuously during the entire decision-making process.

Among the chosen set of criteria, the sustainability criterion requires more attention (Orzes and Sarkis 2019). This becomes evident as a limited number of case studies have focused on sustainability when relocation decisions are being made (Ashby 2016). The case study descriptions in the articles that were coded in the sustainability criterion were limited in comparison to those retrieved for other criteria. Consequently, the retrieved case study descriptions were not sufficient to trigger any improvement of sustainability in relation to relocation. This had a negative impact on the model as the sustainability criterion did not contain any linguistic values other than *neutral* in any of the relocation alternatives outlined in this study. This indicates that sustainability is not sufficiently reflected upon in the relocation decision-making process, which is also in line with the findings reported in the literature (Gray et al. 2017; Pal et al. 2018). Based on the industry experts’ evaluation of the criteria and the case studies from the relocation literature, the sustainability criterion alone is inadequate to form a business case for any type of relocation decision. There is still a need for case studies that can provide rich descriptions about the impacts of relocations triggered by the sustainability criterion. The consequence of this lack of variation in the linguistic labels related to sustainability could also be observed in the sensitivity analysis. During the sensitivity analysis, a horizontal line was observed in all of the relocation scenarios for the sustainability criterion. This implies that the varying importance of the sustainability criterion does not influence the preference for the relocations for the input data.

From the chosen set of criteria, the importance of the quality, time and cost criteria has been sufficiently explored in the relocation literature (Johansson and Olhager 2018). These criteria received high weights compared with the other three criteria. This suggests that quality, time and cost are important criteria to consider in relocation decisions, irrespective of where the production facility is currently situated and where it could be transferred. Previous MCDM models have explored several sub-criteria of the cost and the quality criteria (Kaur et al. 2019; Wei et al. 2019). This also becomes evident in previous case studies that have sufficiently addressed the impacts on the quality, time, and cost criteria during relocations. Therefore, the scenario table (Table 3) consisted of a broader spectrum of linguistic values assigned to the criteria for the different relocations. The sensitivity analysis also reflected the importance of the cost and the quality criteria regarding the relocation preference. During the sensitivity analysis, the highest instability in rankings was observed when the importance of the quality, time and cost criteria was within the range of values from 0 to 0.6, depending on the type of relocation. This implies that the quality, time and cost criteria have a strong influence on the choice of the final relocation alternative.

The sensitivity analysis was performed with the intention of evaluating the stability of the model. This was done by incrementing the weight of each of the six criteria and performing a fuzzy-TOPSIS analysis in an iterative manner. The available relocation alternatives depend on the quadrant showing where a manufacturing company conducts its production operations. For example, if a company currently undertakes its manufacturing activities in Quadrant 1, when cost has very high importance, in-house offshoring and outsourced offshoring relocation are highly preferred to be further evaluated. This is in line with previous research stating that when companies face intense competition based on cost, then offshoring and outsourcing are the most likely scenarios (Gray et al. 2017). Furthermore, this confirms previous findings that either offshoring or outsourcing improves the cost criterion (Di Mauro et al. 2018). In contrast, when manufacturing facilities are located in Quadrant 4 and when cost has low importance, then reshoring for insourcing and outsourced reshoring become interesting. Moreover, when non-cost criteria have high importance, then reshoring or insourcing relocations could be considered. This also aligns with previous findings that when competition is not cost-driven, then companies are more likely to reshore (Gray et al. 2017).

## Conclusions

There is a dearth of decision-support models that aid decision makers in the evaluation of right-shoring decisions. This paper addresses this important gap with respect to decision-making issues when various relocation scenarios are presented as alternatives, something that increases the complexity of the relocation decision. In this research, the developed hybrid fuzzy-AHP-TOPSIS model satisfies the requirement of incorporating both uncertainty and heuristics in a relocation decision. Relocation scenarios were generated by coding case study descriptions and findings from the relocation literature. The developed model provides an importance rating of the decision criteria and evaluates the relocation alternatives, returning a ranking of the alternatives that a company needs to evaluate further. The usability of the model is further improved by presenting a sensitivity analysis that provides a ranking of the alternatives when the importance of the criteria is either increasing or decreasing. Both the model and sensitivity analysis were implemented using Excel and Python. The study indicates that the six holistic criteria that are used in the model are appropriate to consider when evaluating the relocation scenarios, of which the cost, quality and time criteria are the most important ones, while more research is required to fully understand the impact of relocations on the sustainability criterion.

This research contributes to theory and practice in several ways. First, it enriches the ongoing debate on right-shoring decisions by considering multiple relocation scenarios with governance modes in the decision-making process. As previous research has focused on a limited view on relocation, that is, offshoring (and/or outsourcing) versus reshoring (and/or insourcing), this research combines the main scenarios in the relocation decision-making process. Furthermore, there is a need to develop decision-support models for reshoring decisions; thus, this research also contributes with a fuzzy-MCDM model for right-shoring decisions. The resilience of the decision-support model is addressed through a sensitivity analysis in this research, suggesting that the choice of the preferred scenario depends on the importance of the decision criteria. Among the six competitive priorities’ criteria, cost, quality and time have a strong influence on the choice of the final relocation alternative. This research further contributes to the literature on MCDM methods with a hybrid fuzzy-AHP-TOPSIS model that combines the advantage of the simplicity of AHP with the scalability of TOPSIS to reach a right-shoring decision.

This research makes several practical contributions. It provides managers with a decision-support model to evaluate their relocation scenarios. Specifically, this model has been used in the scenario generation and evaluation steps of the relocation decision-making process. However, it can be further applied to initial screening, as well when a company should be able to make an early decision on whether or not to continue the relocation evaluation process, possibly saving both its time and resources. Another contribution to practice is that the sensitivity analysis in the model allows practitioners to make resilient relocation decisions. Practitioners need to be aware of the lines crossing that triggers a different scenario. The criteria where too many lines cross need to be scrutinized. In cases where the lines do not cross, the weight of the criteria do not trigger a different scenario. This research further supports managers in making the correct long-term decision with the help of the presented model.

For future research, an important step would be to investigate the impact of various relocations on the sustainability criterion. A limited number of studies focus exclusively on the impact on sustainability, despite the availability of some support model for making sustainable choices in the supply chain. Therefore, some case studies on the sustainability impact would further increase the understanding of this criterion. Another avenue for future research would be to generate relocation scenarios in an empirical manner by conducting a single longitudinal case study. Companies are expected to encounter more than one scenario for each relocation type, different from this study, where only one scenario for each relocation type was developed. Future scenarios must also encompass the different suppliers or facilities that are considered in the decision-making stage. In this study, case study descriptions from the literature were used for generating relocation scenarios. With the advancement in machine learning applications, natural language processing tools could be used to generate relocation scenarios automatically from a large body of the relocation literature.

## Appendix

### Appendix 1: Classification of expertise in the manufacturing company

**Table:**

**Expert**	**Competences**	**Role in company**	**Experience in company**
Expert A	Production engineering, Lean production	Site Manager (Sweden)	4 years
Expert B	Change management, Mergers and Acquisition	HR Director (Nordic region)	5 years
Expert C	Production management, Lean	Industrial Engineering Manager	9 years
Expert D	Production Management	Operations Manager	20 years
Expert E	Sourcing, Material handling	Purchasing Manager	9 years

### Appendix 2: Literature search process and sample generation

**Table:**

**Search strings**	**Time**	**Document type**	**Search process**	**Total**	**Note**
TITLE-ABS-KEY ((reshoring OR backshoring OR offshoring OR insourcing OR outsourcing) AND ("case stud*") AND (manufacturing))	2000–2020	Article or Review	Sample after search	235	
			**Title-abstract-keywords review**		
			Sample after TITLE-ABS-KEY screening	75	Other topics were excluded (e.g., environmental science, marine engineering, among others)
			Exclusion criteria (Relocation decision already taken OR case study involves a previous decision)	59	Excluded paper that have not yet made decision (e.g., development of decision support)
			**Full paper review**		
			Exclusion criteria (Case description not sufficient to map location and sourcing dimensions)	30	Excluded papers that have not provided case description detailed enough to map the location and sourcing dimensions
			Snowballing	2	Unmapped relocations were searched using snowballing technique to include Bals et al. 2016; Gray et al. 2017
			Final sample	32	

### Appendix 3: Classification of cases from final sample

**Table:**

**#**	**Case**	**Relocation**	**Source**	**Cost**	**Quality**	**Time**	**Flexibility**	**Innovation**	**Sustainability**
1	Aku	1- Reshoring of in-house activity	Di Mauro et al. (2018)	0	1	1	1	4	0
2	FurnitureCo	1- Reshoring of in-house activity	Engström et al. (2018b)	1	2	2	2	0	1
3	ClothingCo	1- Reshoring of in-house activity	Engström et al. (2018b)	0	4	1	1	1	0
4	TruckCo	1- Reshoring of in-house activity	Engström et al. (2018b)	0	2	2	1	0	1
5	Company A	1- Reshoring of in-house activity	Sayem et al. (2019)	0	3	3	0	0	0
6	Auto	1- Reshoring of in-house activity	Lund and Steen (2020)	3	0	0	1	2	0
7	Construction	1- Reshoring of in-house activity	Lund and Steen (2020)	2	0	0	0	3	0
8	Alpha	1- Reshoring of in-house activity	Stentoft et al. (2016a)	1	0	0	1	0	0
9	Fitwell	2- Reshoring for insourcing	Di Mauro et al. (2018)	-1	4	0	0	1	0
10	Roncato	2- Reshoring for insourcing	Di Mauro et al. (2018)	0	4	0	0	1	0
11	Ska Italia	2- Reshoring for insourcing	Di Mauro et al. (2018)	0	3	0	1	2	0
12	FixingsCo	2- Reshoring for insourcing	Engström et al. (2018b)	-2	1	2	2	0	0
13	Company B	2- Reshoring for insourcing	Sayem et al. (2019)	4	3	1	0	0	0
14	Offshore	2- Reshoring for insourcing	Lund and Steen (2020)	2	0	1	0	2	0
15	Aqua	2- Reshoring for insourcing	Lund and Steen (2020)	1	0	2	0	2	0
16	Marine	2- Reshoring for insourcing	Lund and Steen (2020)	3	0	0	0	1	0
17	Maritime	2- Reshoring for insourcing	Lund and Steen (2020)	1	2	0	1	0	0
18	Autoco	2- Reshoring for insourcing	Moradlou et al. (2020)	0	1	1	1	0	0
19	Rubberco	2- Reshoring for insourcing	Moradlou et al. (2020)	1	1	2	0	0	0
20	Garmentco	2- Reshoring for insourcing	Moradlou et al. (2020)	0	1	1	1	1	0
21	Sofaco	2- Reshoring for insourcing	Moradlou et al. (2020)	1	0	2	0	0	0
22	Bedco	2- Reshoring for insourcing	Moradlou et al. (2020)	1	0	1	1	0	0
23	Computerco	2- Reshoring for insourcing	Moradlou et al. (2020)	1	0	1	0	1	0
24	Burberry	2- Reshoring for insourcing	Robinson and Hsieh (2016)	0	4	2	0	0	0
25	Company E	2- Reshoring for insourcing	Joubioux and Vanpoucke (2016)	1	2	0	0	0	0
26	Company F	2- Reshoring for insourcing	Joubioux and Vanpoucke (2016)	1	1	0	0	0	0
27	Case A	2- Reshoring for insourcing	Hartman et al. (2017)	1	1	0	0	0	0
28	Case D	2- Reshoring for insourcing	Hartman et al. (2017)	1	1	0	0	0	0
29	Case K	2- Reshoring for insourcing	Hartman et al. (2017)	1	1	0	0	0	0
30	Case L	2- Reshoring for insourcing	Hartman et al. (2017)	1	1	0	0	0	0
31	Clothing SME	3- Reshoring of outsourced activity	Ashby (2016)	0	3	0	4	2	6
32	SME A	3- Reshoring of outsourced activity	Gray et al. (2017)	0	2	1	1	0	0
33	SME B	3- Reshoring of outsourced activity	Gray et al. (2017)	2	5	0	3	1	0
34	SME C	3- Reshoring of outsourced activity	Gray et al. (2017)	0	6	1	2	0	0
35	SME D	3- Reshoring of outsourced activity	Gray et al. (2017)	0	0	0	0	0	1
36	Company C	3- Reshoring of outsourced activity	Sayem et al. (2019)	2	4	3	0	0	0
37	Telecom	3- Reshoring of outsourced activity	Lund and Steen (2020)	2	0	2	1	0	0
38	Company A	3- Reshoring of outsourced activity	Bettiol et al. (2017)	0	2	1	0	0	0
39	Company B	3- Reshoring of outsourced activity	Bettiol et al. (2017)	0	2	0	0	0	0
40	Fitwell	3- Reshoring of outsourced activity	Baraldi et al. (2018)	0	2	1	0	0	0
41	Solari	4- Reshoring for outsourcing	Grandinetti and Tabacco (2015)	0	0	7	0	6	0
42	JP Morgan Chase	5- Offshoring for insourcing	Bals et al. (2016)	0	0	0	0	1	0
43	Walmart	5- Offshoring for insourcing	Bals et al. (2016)	0	0	0	1	0	0
44	Velox	6- Offshoring for outsourcing	Gylling et al. (2015)	5	-2	-3	-4	0	0
45	Fitwell	6- Offshoring for outsourcing	Di Mauro et al. (2018)	7	2	0	1	0	0
46	Thomas	6- Offshoring for outsourcing	Buciuni et al. (2014)	1	0	0	0	0	0
47	Bern	6- Offshoring for outsourcing	Buciuni et al. (2014)	1	0	0	0	0	0
48	Catawba	6- Offshoring for outsourcing	Buciuni et al. (2014)	0	0	0	1	0	0
49	Company A	6- Offshoring for outsourcing	Joubioux and Vanpoucke (2016)	0	0	-1	0	0	0
50	Company B	6- Offshoring for outsourcing	Joubioux and Vanpoucke (2016)	0	0	0	0	0	0
51	Company C	6- Offshoring for outsourcing	Joubioux and Vanpoucke (2016)	1	1	-1	0	0	0
52	Company D	6- Offshoring for outsourcing	Joubioux and Vanpoucke (2016)	-1	0	0	0	0	0
53	Company E	6- Offshoring for outsourcing	Joubioux and Vanpoucke (2016)	-1	-2	0	0	0	0
54	Company F	6- Offshoring for outsourcing	Joubioux and Vanpoucke (2016)	0	-2	-1	0	0	0
55	Fitwell	6- Offshoring for outsourcing	Baraldi et al. (2018)	3	0	1	2	0	0
56	Case A	6- Offshoring for outsourcing	Dekkers (2011)	-2	-3	-4	0	0	0
57	Case B	6- Offshoring for outsourcing	Dekkers (2011)	-1	-1	-3	1	0	0
58	Case C	6- Offshoring for outsourcing	Dekkers (2011)	0	-2	-2	1	0	0
59	Case D	6- Offshoring for outsourcing	Dekkers (2011)	1	-2	-1	-1	0	0
60	Case E	6- Offshoring for outsourcing	Dekkers (2011)	-1	-6	-1	0	0	0
61	Case F	6- Offshoring for outsourcing	Dekkers (2011)	-1	-1	-1	0	0	0
62	Case G	6- Offshoring for outsourcing	Dekkers (2011)	-2	-2	-1	0	0	0
63	Auto	6- Offshoring for outsourcing	Mohiuddin and Su (2013)	3	0	0	0	0	0
64	Ceramic	6- Offshoring for outsourcing	Mohiuddin and Su (2013)	1	-1	0	0	0	0
65	Electric	6- Offshoring for outsourcing	Mohiuddin and Su (2013)	3	0	0	0	0	0
66	Electronic	6- Offshoring for outsourcing	Mohiuddin and Su (2013)	1	1	0	0	0	0
67	Furniture	6- Offshoring for outsourcing	Mohiuddin and Su (2013)	2	0	0	0	0	0
68	Garment	6- Offshoring for outsourcing	Mohiuddin and Su (2013)	2	1	0	0	0	0
69	High-tech	6- Offshoring for outsourcing	Mohiuddin and Su (2013)	1	0	0	0	0	0
70	IE	6- Offshoring for outsourcing	Mohiuddin and Su (2013)	1	0	0	0	0	0
71	Shoe	6- Offshoring for outsourcing	Mohiuddin and Su (2013)	1	-1	0	0	0	0
72	Tools	6- Offshoring for outsourcing	Mohiuddin and Su (2013)	2	0	0	0	0	0
73	Case A	6- Offshoring for outsourcing	Nordigården et al. (2014)	2	1	0	2	0	0
74	C1	6- Offshoring for outsourcing	Sinha et al. (2011)	-1	0	0	0	0	0
75	C2	6- Offshoring for outsourcing	Sinha et al. (2011)	1	0	-1	0	0	0
76	C3	6- Offshoring for outsourcing	Sinha et al. (2011)	1	2	-1	1	0	0
77	C4	6- Offshoring for outsourcing	Sinha et al. (2011)	2	1	0	0	-1	0
78	C5	6- Offshoring for outsourcing	Sinha et al. (2011)	2	-1	0	0	0	0
79	Firm A	6- Offshoring for outsourcing	Yu and Lindsay (2011)	0	0	-1	-1	0	0
80	Firm B	6- Offshoring for outsourcing	Yu and Lindsay (2011)	0	0	-1	-2	0	0
81	Firm C	6- Offshoring for outsourcing	Yu and Lindsay (2011)	2	0	1	1	0	0
82	Firm D	6- Offshoring for outsourcing	Yu and Lindsay (2011)	2	1	-1	-2	0	0
83	Firm E	6- Offshoring for outsourcing	Yu and Lindsay (2011)	3	0	-3	-1	0	0
84	Firm F	6- Offshoring for outsourcing	Yu and Lindsay (2011)	0	0	-2	0	0	0
85	Apptech	6- Offshoring for outsourcing	Zhang et al. (2013)	-1	0	1	0	0	0
86	Chem Ltd	6- Offshoring for outsourcing	Zhang et al. (2013)	2	0	1	-1	0	0
87	Pharsol	6- Offshoring for outsourcing	Zhang et al. (2013)	0	0	0	1	0	0
88	Syne Ltd	6- Offshoring for outsourcing	Zhang et al. (2013)	1	1	1	2	-1	0
89	Company A	7- Offshoring of outsourced activity	Song N., Platts K., Bance D	-1	-2	0	0	0	0
90	Aku	8- Offshoring of in-house activity	Di Mauro et al. (2018)	3	4	1	0	0	0
91	FixingsCo	8- Offshoring of in-house activity	Engström et al. (2018b)	1	0	0	0	0	0
92	Alpha	8- Offshoring of in-house activity	Stentoft et al. (2016b)	2	-1	0	-2	0	0
93	Case 1	8- Offshoring of in-house activity	Arlbjørn and Lüthje (2012)	6	3	6	0	0	0
94	Case 2	8- Offshoring of in-house activity	Arlbjørn and Lüthje (2012)	-2	0	0	0	0	0
95	Company B	8- Offshoring of in-house activity	Jørgensen et al. (2015)	-1	-1	0	0	0	0
96	Case study 1	9- Insourcing of domestic activity	Drauz (2014)	1	0	0	2	0	0
97	Case study 2	9- Insourcing of domestic activity	Drauz (2014)	0	0	0	1	0	0
98	Case study 3	9- Insourcing of domestic activity	Drauz (2014)	1	0	0	1	1	0
99	Case study 4	9- Insourcing of domestic activity	Drauz (2014)	0	0	1	1	1	0
100	Case study 5	9- Insourcing of domestic activity	Drauz (2014)	2	1	0	2	0	0
101	Case study 6	9- Insourcing of domestic activity	Drauz (2014)	1	0	0	2	0	0
102	Firm F	9- Insourcing of domestic activity	Hartman et al. (2017)	1	1	0	0	0	0
103	Firm G	9- Insourcing of domestic activity	Hartman et al. (2017)	1	0	0	0	0	0
104	Firm H	9- Insourcing of domestic activity	Hartman et al. (2017)	1	1	0	0	0	0
105	Apparel	10- Insourcing of offshored activity	Caputo and Palumbo (2005)	5	2	1	5	0	0
106	Klamath	11- Outsourcing of domestic activity	Buciuni et al. (2014)	0	0	1	1	0	0
107	Elm	11- Outsourcing of domestic activity	Buciuni et al. (2014)	0	2	1	1	0	0
108	Kährs	11- Outsourcing of domestic activity	Rehme et al. (2013)	0	0	0	1	0	0
109	Elitfönster	11- Outsourcing of domestic activity	Rehme et al. (2013)	0	-1	0	-2	0	0
110	Alpha	12- Outsourcing of offshored activity	Nujen et al. (2018)	2	-2	0	0	-5	0
111	Beta	12- Outsourcing of offshored activity	Nujen et al. (2018)	0	0	0	0	-2	0
112	Delta	12- Outsourcing of offshored activity	Nujen et al. (2018)	1	0	0	-1	0	0
113	Aalborg Industries	12- Outsourcing of offshored activity	Momme and Hvolby (2002)	1	0	0	0	0	0
114	Case 3	12- Outsourcing of offshored activity	Arlbjørn and Lüthje (2012)	4	0	1	0	-1	0
115	Case 4	12- Outsourcing of offshored activity	Arlbjørn and Lüthje (2012)	-1	-1	-1	0	0	0
